# Modeling of 2D diffusion processes based on microscopy data: parameter estimation and practical identifiability analysis

**DOI:** 10.1186/1471-2105-14-S10-S7

**Published:** 2013-08-12

**Authors:** Sabrina Hock, Jan Hasenauer, Fabian J Theis

**Affiliations:** 1 Institute of Computational Biology, Helmholtz Center Munich, Ingolstädter Landstr. 1, 85764 Neuherberg, Germany; 2 Department of Mathematics, Technische Universität München, Boltzmannstr.3, 85747 Garching, Germany

## Abstract

**Background:**

Diffusion is a key component of many biological processes such as chemotaxis, developmental differentiation and tissue morphogenesis. Since recently, the spatial gradients caused by diffusion can be assessed in-vitro and in-vivo using microscopy based imaging techniques. The resulting time-series of two dimensional, high-resolutions images in combination with mechanistic models enable the quantitative analysis of the underlying mechanisms. However, such a model-based analysis is still challenging due to measurement noise and sparse observations, which result in uncertainties of the model parameters.

**Methods:**

We introduce a likelihood function for image-based measurements with log-normal distributed noise. Based upon this likelihood function we formulate the maximum likelihood estimation problem, which is solved using PDE-constrained optimization methods. To assess the uncertainty and practical identifiability of the parameters we introduce profile likelihoods for diffusion processes.

**Results and conclusion:**

As proof of concept, we model certain aspects of the guidance of dendritic cells towards lymphatic vessels, an example for haptotaxis. Using a realistic set of artificial measurement data, we estimate the five kinetic parameters of this model and compute profile likelihoods. Our novel approach for the estimation of model parameters from image data as well as the proposed identifiability analysis approach is widely applicable to diffusion processes. The profile likelihood based method provides more rigorous uncertainty bounds in contrast to local approximation methods.

## Introduction

Diffusion is assumed to be the basis of many spatial organization processes for multi-cellular organisms. Crucial processes such as developmental pattern formation or chemotaxis rely on gradient information arising from diffusion and transport processes [1,2]. In the last decades, diffusion processes have been of great interest not only for experimentalist but also for theoreticians. Turing [3] was the first to break ground, followed by Gierer and Meinhardt [4], who introduced models for such processes based on partial differential equations (PDEs). A prominent aspect is the diffusion of extracellular signaling molecules. Such molecules are synthesized and secreted by cells and spread through the surrounding tissue, forming a gradient. A biological prominent example is guided cell movement along such gradients. In this case, the cell senses the concentration difference between front and back, and moves along the gradient.

**Figure 1:**
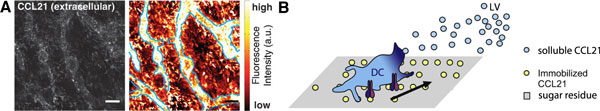
**Haptotaxis: Data and schematic description of the process**. Haptotaxis: Data and schematic description of the process. (A) Fluorescence staining image taken from [7], which shows the Z-stack projection of non-permeabilized ear dermis stained for CCL21. Left image is the maximum intensity projection and the right image shows same staining as color-coded average projection. Lymphoid vessel boundaries are indicated by the blue dotted line (scale bars: 100*µm*). (B) Schematic of the dendritic haptotaxis process adapted from [6]. Dendritic cells move along a gradient of immobilized CCL21 towards the lymphatic vessels.

Gradients of signaling molecules can be made visible in-vivo via antibody stainings (see Figure 1 and [5-7]). Combined with microscopy, this yields two-dimensional (2D) images. The color intensity of each pixel provides informations about the concentration (or the number) of signaling molecules. Modern microscopy devices can also generate stacks of images, providing information about the distribution of signaling molecules in three-dimensions (3D) [5,8].

Despite these high-resolution imaging data, the number of quantitative models of biological diffusion processes is limited. While quantitative modeling with ordinary differential equations (ODEs) is a common method and the theory of parameter estimation and identifiability is sound, those results have yet to be transferred to the quantitative modeling with PDEs [9,10]. In recent years the field of PDE-constrained optimization emerged, providing the theory and methods to estimate parameters of PDEs [11]. Nevertheless, specific problems occurring in biological problems, like partial observations, sparse measurements and high noise levels, have yet to be addressed. This has already been done for ODE parameter estimation techniques [12] but is an open problem in the PDE context. In particular, appropriate likelihood functions and methods for the efficient and reliable analysis of practical identifiability [9] are not available.

In this paper, we propose a likelihood function for the estimation of parameters of 2D diffusion process from image data. Furthermore, we transfer the concept of profile likelihood based identifiability analysis introduced by Raue et al. [9] from ODEs to PDEs. This allows us to go beyond the classical uncertainty analysis methods based on local approximation towards global uncertainty bounds. Finally, we evaluate the methods by studying a model for diffusion processes involved in the migration of dendritic cells towards lymphatic vessels (see schematic picture Figure 1B).

## Methods

In the following section we shortly introduce the considered class of PDEs and the available types of measurement data. Afterwards, the parameter estimation and identifiability analysis methods are presented.

### Problem description

For *t *∈ (0, *T*], $x\in \text{Ω}\subset {\mathbb{R}}^{2}$ and $\varphi \in {\mathbb{R}}_{+}^{n_{\varphi }}$ we consider reaction-diffusion models of the form

(1) $$\frac{\partial }{\partial t}u\left(t,x\right)-D\text{Δ}u\left(t,x\right)=f\left(t,x,u,\varphi \right),$$

where *u*(*t*, *x*) is a vector-valued function describing concentrations, molecule numbers or similar entities for a set of interacting substances. For non-diffusive components the corresponding entries of the diagonal diffusion matrix *D *are zero. The model is complemented with boundary conditions and initial conditions. In the following, we assume that boundary conditions, initial conditions and *f *(*t*, *x*, *u*, *φ*) are chosen such that for all *x*, *t *and *φ *a unique, integrable (with respect to *x*) solution *u*(*t*, *x*) exists in an appropriate function space *U *.

In many cases the spatial and temporal behavior of reaction-diffusion processes is studied by means of image data collected using microscopy devices. We consider a time-series of images taken at time points *t_k _*for *k *= 1, . . . , *N*, which are not necessarily equally spaced. For each image the number of pixels *M *and their pixel area $A_{p_{t}}$ is known. A suitable function to map the state *u*(*t*, *x*; *φ*) to the observables is

(2) $$y_{k,i}\left(u,\varphi \right)=b+\int_{A_{p_{{}_{i}}}}h\left(u\left(t_{k},x;\varphi \right)\right)\;dx$$

for *i *= 1, . . . , *M *and *k *= 1, . . . , *N *. Here $b\in {\mathbb{R}}_{+}$ denotes a constant off-set due to background luminescence and *h *defines the observables and could for instance be a mapping onto the first component of *u*. With our assumptions made about existence, uniqueness and integrability this is a well-defined function.

Biological measurement data are in general noise corrupted. The noise distribution depends on the measurement techniques. As measured fluorescence intensities are always positive and as image acquisition is basically a counting process, we assume multiplicative log-normal measurement noise, i.e.

(3) $${ȳ}_{k,i}=\varepsilon y_{k,i}.$$

With ${ȳ}_{k,i}$ we denote the intensity of pixel *i *at time point *t_k_*. We assume that $\varepsilon \sim LN\left(0,\sigma^{2}\right)$, hence ${ȳ}_{k,i}\sim LN\left(y_{k,i},\sigma^{2}\right)$. In the following, we introduce the corresponding likelihood function which is used to estimate the unknown parameters *θ *= (*φ*, *b*, *σ*^2^).

### Maximum likelihood estimation

For multiplicative log-normal noise the likelihood function is

(4) $$L\left(\theta \right)=\underset{k,i}{\prod }\frac{1}{\sqrt{2\pi }\sigma {ȳ}_{k,i}}\text{exp}\left(-\frac{1}{2}{\left(\frac{\text{log}\left({ȳ}_{k,i}\right)-\text{log}\left(y_{k,i}\right)}{\sigma }\right)}^{2}\right).$$

The statistically most consistent parameters are those, which maximize the likelihood function, i.e. the maximum likelihood estimator (MLE). Instead of maximizing the likelihood function commonly the negative logarithm of the likelihood function, *J*(*θ*) = *− *log *L*(*θ*), is minimized to improve the numerics. The minimization problem for parameter estimation for models of the type (1) is then given as

(5) θ∗=arg min(u,θ)∈U×ℝ+nθJ(θ)         s.t. yk,i(u)=b+∫Apih(u(tk,x)) dx             ∂∂tu(t,x)−DΔu(t,x)=f(t,x,u,φ).

Optimization problems of this type belong to the class of PDE-constrained optimization problems, for which different numerical methods have been established (see [11] and references therein). Depending on the problem structure the PDE is either first optimized and then discretized or discretized and the optimized, which is often necessary and can be justified mathematically [11]. For the example considered, we used the second approach. Furthermore, we optimize the logarithm of the parameters instead of the parameters themselves. This take care of the natural positivity constraints and has for ODE models been shown to be more reliable.

While the optimization problem (5) can be solved numerically, the main problem for parameter estimation is the shape of the likelihood function. Non-identifiabilities and non-linear correlated parameters, leading to 'banana-shaped' likelihoods, render local approximation methods for the evaluation of confidence intervals often inaccurate.

### Profile likelihood based identifiability analysis

The uncertainty of the MLE is commonly analyzed by a local approximation of the objective function and the resulting asymptotic confidence intervals. This local approximation, however, is not reliable for nonlinear problems when we are interested global uncertainty bounds.

The profile likelihood (PL) is a tool to quantify the uncertainty of the MLE and to determine global uncertainty bounds, therefore, the MLE is calculated for a one-dimensional sub-space of the parameter space. In our case we calculate the profile likelihood for the unknown parameters of interest, i.e. *θ_i_*. For parameter *θ_i_*, PL(*θ_i_*) is computed by the re-optimization of all parameters *θ_j _≠ θ_i _*along the profile of parameter *θ_i _*[13]:

(6) $$\text{PL}(\theta_{i})=\underset{\theta_{j\neq i}}{\max L(\theta )}=\exp\left(-\underset{\theta_{j\neq i}}{\min}J(\theta )\right).$$

The minimization must fulfill the same constraints as in (5). This can be repeated for all parameters *θ_i_*, *i *= 1, . . . , *n_θ_*, and allows the evaluation of the likelihood ratio *R*(*θ_i_*) = PL(*θ_i_*)*/L*(θ*) for the individual parameters. Based on the likelihood ratio *R*(*θ_i_*) we can determine globally valid confidence intervals for the parameter *θ_i_*,

$$C_{i}=\left\{\theta_{i}\left|R\left(\theta_{i}\right)<\text{exp}\left(-\frac{\delta_{\alpha }}{2}\right)\right)\right\},$$

with confidence level *α *and the corresponding likelihood ratio threshold *δ_α _*= *χ*^2^(*α*, 1) [13]. And according to [9] a parameter is called practical non-identifiable if the likelihood ration does not fall below the threshold *δ_α _*for increasing and decreasing values of *θ_i_*. Hence a profile likelihood which is flat, i.e. remains above the threshold *δ_α_*, indicates a practically unidentifiable parameter. For systems of ordinary differential equations the profile likelihood calculation has been shown to be a suitable method to quantify the practical identifiability and the uncertainty of parameters.

## Results

To illustrate the proposed parameter estimation and uncertainty analysis framework, we consider the formation of gradients of signaling protein which are immobilized by the extracellular matrix. Such gradients are, for instance, the basis of the haptotaxis of dendritic cells towards the lymphatic vessels upon the detection of unknown antigenes [6,7] (see Figure 1B). In this process, dendritic cells move towards the closest lymphatic vessel in the tissue and are subsequently transported through the lymphoid system towards the lymph nodes. The movement of the dendritic cells is guided by an immobilized gradient of the cytokine CCL21, which is released from the lymphoid vessels [6].

In the following we formulate a model for the gradient formation process. In our model, the signaling protein CCL21, denoted by *P*, is produced constantly at a spatially distributed source *Q*, the lymphoid system. The signaling protein gradient is immobilized through complex formation with a tissue bound sugar, denoted by *S*. The immobilized CCL21 protein is denoted by *C*.

Following the problem formulation, we study a process containing three state variables: *p*, *s *and *c*. Each variable is a function of the spatial location *x *∈ Ω = [0,1]^2^, time *t *∈ [0, *T*] and a set of unknown parameters *θ*. The model considered is:

(7) $$\begin{array}{c} \;\frac{\partial }{\partial t}p\;-\;D\text{Δ}p=\alpha Q-k_{1}ps+{k_{-}}_{1}c-\gamma p\\ \;\;\;\;\;\frac{\partial }{\partial t}s=-k_{1}ps+k_{-1}c\\ \frac{\partial }{\partial t}c=k_{1}ps-k_{-1}c \end{array}$$

for *t *∈ (0, *T*] and *x *∈ Ω, with initial conditions

(8) $$\forall x\in \Omega :p\left(0,x\right)=c\left(0,x\right)=0\text{ and }s\left(0,x\right)=s_{0},$$

and no-flux boundary conditions,

(9) $$\frac{\partial }{\partial \nu }p=0,$$

where *ν *denotes the outer normal of Ω. The binding and unbinding rates of CCL21 and tissue-bound sugar are denoted by *k*_1 _and *k*_*−*1_. The diffusion constant of CCL21, the rate of CCL21 degradation and the rate of CCL21 release from the lymph system are *D*, *γ *and *α*, respectively. In the following, we assume that *Q *: Ω *→ *{0, 1} and *s*_0 _*≡ *1, due to scaling. We consider the kinetic parameters *θ *= (*D*, *α*, *k*_1_, *k*_*−*1_, *γ*) of this model as unknowns with *θ *∈ [10^*−*2^, 10^1^]^5^.

For this process image-resolved measurements of the immobilized CCL21 have been taken at one time point (see Figure 1A and [7]). And it might be assumed that the process has reached a steady state (personal communication with authors of [7]). Analytical analysis of the model (1) showed that not all parameters are identifiable from steady state data. In particular, it can be shown that the reaction rates *k*_1 _and *k*_*−*1 _are structurally not identifiable. In the following, we want to analyze whether time-series data are sufficient to estimate all kinetic parameters of the model. We want to address this question with the image-based profile likelihood method introduced above and the model considering signaling protein, substrate and substrate-bound protein.

The measured output of the system (7)-(9) are microscopy images of tissue stained for the complex *C*. According to (2) we have

$$y_{i,k}=b+\int_{A_{p_{{}_{i}}}}c\left(t_{k},x\right)\;dx.$$

**Figure 2:**
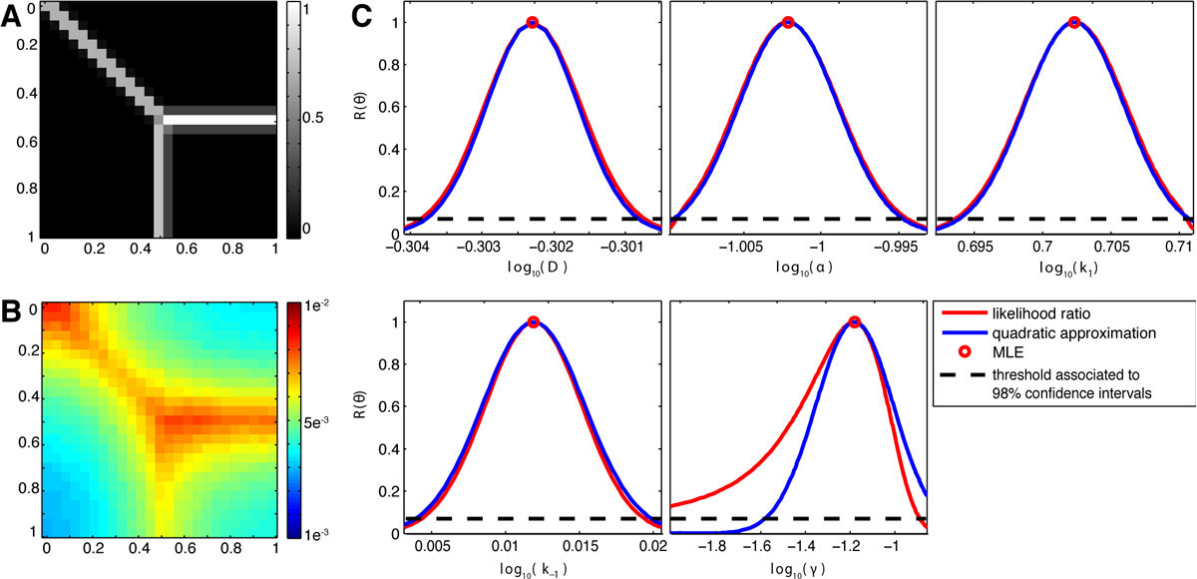
**Parameters estimation for the diffusion model**. (A) Shows the source term *Q *for an early time point. (B) Shows ȳ  for the last time point *t*_5_. (C) Likelihood ratio calculated for the five dynamic parameters *D*, *α*, *k*_1_, *k*_−1 _and *γ *are shown in red. The second-order local approximation used for asymptotic confidence intervals is given in blue. The x-axis is given as the logarithm of the parameters, which was also used for the estimation process.

To calculate the output function we discretized (7)-(9) by finite differences and numerically integrated the discretized state variable *c*. For the estimation process, data are generated via model simulation (for parameters see Table 1). Forthese simulations we chose a y-shape source term imitating a lymphoid vessel branch (see Figure 2A). We consider images taken at five time points *t_k _*∈ (0, 1], *k *= 1, . . . , 5 with 50 pixels each $A_{p_{t}}$, *j *= 1, . . . , 50. To account for measurement noise, log-normal noise was added (according to (3)) with σ = 10^−2^ and *b *= 10^−4^ (see Figure 2B for one representative image).

**Table 1:** Estimation results

Name	value	MLE	parameter bounds		confidence intervals		identifiability
	***θ***	$\hat{\theta }$	***θ_min_***	***θ_max_***	***C*_*i,min*_**	***C*_*i,max*_**	

*D*	0.5	0.4985	0.01	10	0.4939	0.5044	practical identifiable
*α*	0.1	0.0995	0.01	10	0.0969	0.1059	practical identifiable
*k*_1_	5	5.0375	0.01	10	4.6841	5.1761	practical identifiable
*k*_−1 _	1	1.0281	0.01	10	0.9704	1.0687	practical identifiable
*γ*	0.1	0.0669	0.01	10	*<*0.0100	0.3272	**practically non-identifiable**

True value of each estimated parameter compared to maximum likelihood estimate for a local multiple start optimization. Bounds for each parameter where used for estimation.

For this artificial data set the maximum likelihood estimate θ* is shown in Table 1. The chosen data points where sufficient to identify parameters *D*, *α*, *k*_1 _and *k*_−1 _well. They are practical identifiable on a confidence level *α *= 98% as the likelihood ration *R*(*θ*) falls below the given threshold for increasing and decreasing values of the parameters (Figure 2). For these parameters a Hessian based approximation of the likelihood at the ML estimate yields a good approximation of the profile likelihood (Figure 2). This is not the case for *γ*. For *γ*, the Hessian based approximation of the likelihood function underestimates the true uncertainty. Indeed, the profile likelihood for *γ *reveals that the parameter is practical non-identifiable as no lower bound exists in the considered regime. Thus, for this parameter, the analysis of the profile likelihood is required to assess the uncertainty of the parameter estimation.

The identifiability properties as well as the parameter confidence intervals change depending on the noise levels and the number of time points M. Simulation results show that, as expected, the confidence interval width increases then the noise levels increase. Additionally the practical non-identifiability of parameter *γ *increases drastically with the noise level. An increased number of time points results in tighter confidence intervals and improved identifiability properties. If the number of time points is large enough the degradation *γ *even becomes identifiable (results not shown). This shows that with time-resolved data all parameters can be identified.

## Discussion and conclusion

In this paper we introduced profile likelihood-based identifiability analysis for diffusion processes based on 2D image data. As proof of concept, we applied our method to a reaction diffusion system involved in the guidance of dendritic cells to the lymphatic vessels [6,7]. Based on current knowledge this is the first paper using profile likelihood methods in this context.

Our approach facilitates the rigorous definition of uncertainty bounds compared to local approximative methods like the approximation of the Hessian matrix. This allows us to determine precisely which parameters can be identified, which we illustrate for a model describing the formation of CCL21 gradients, involved in the guidance of dendritic cells. Furthermore, profile likelihood-based uncertainty analysis also facilitates the planning of experiments [14]. If a specific parameter of the model is of particular biological interest its expected identifiability properties after performing a proposed experiment can be analyzed. Another strength of the likelihood function introduced is the straight forward extension to voxel based data, i.e. 3D image stacks. In the current setup the 2D area integral in (2) becomes a 3D volume integral. We will address this extension and it's application in future work.

In the illustrative example we used a simple finite difference method to discretize the Laplace operator. This approximation scheme sufficed as we knew the exact parameter values and could set parameter bounds for the optimization such that the discretization errors did not influence the optimization. In real applications this is impossible and an adaptive scheme has to be applied to ensure convergence of the PDE solver [11]. Otherwise the profile likelihood calculation is affected by discretization errors and no longer reliable.

Our analysis of the illustrative example showed that time-series image-data are particularly suitable to estimate the kinetic parameters of a reaction-diffusion processes. An interesting question for future work is whether dose response experiments yield similar results. Finally, the profile likelihood analysis yields a more reliable estimate of the uncertainty in the parameter estimation for such processes and is required to give rigorous global uncertainty bounds. Given the introduced likelihood function we could now approach the model selection in a statistical reasonable way.

## Competing interests

The authors declare that they have no competing interests.

## Authors' contributions

SH and JH designed the method and performed the calculations. JH and FJT devised and coordinated the project. All authors contributed to the paper writing.

## Declarations

Publication costs were financed by the Helmholtz Zentrum Muenchen GmbH.

This article has been published as part of BMC Bioinformatics Volume 14 Supplement 10, 2013: Selected articles from the 10th International Workshop on Computational Systems Biology (WCSB) 2013: Bioinformatics. The full contents of the supplement are available online at http://www.biomedcentral.com/bmcbioinformatics/supplements/14/S10.
